# Supplementary material for: Dynamic weakening during earthquakes controlled by fluid thermodynamics
Source: Nat Commun. 2018 Aug 6;9:3074. doi: 10.1038/s41467-018-05603-9 (PMC6079085; doi:10.1038/s41467-018-05603-9)
Supplement: Supplementary file 1 — Supplementary Information [file 41467_2018_5603_MOESM1_ESM.pdf]

Supplementary information for:

Dynamic weakening during earthquakes controlled by fluid thermodynamics

By M.Acosta* et al.

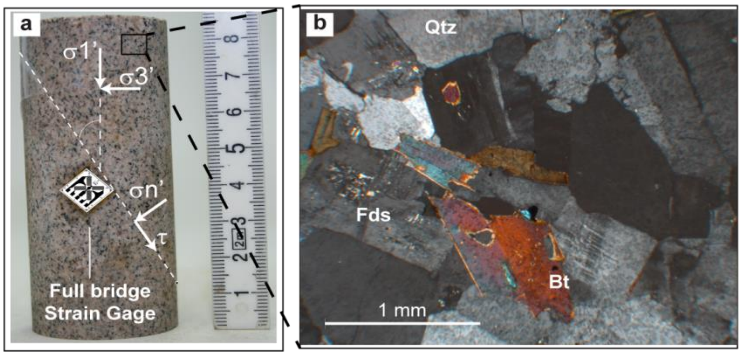

Supplementary Figure 1. Experimental Sample.

(a) Experimental sample of Westerly Granite (WG); cylinder configuration. Grain size is inferior to 1 mm, material is homogeneous and isotropic. The artificial fault is marked as a dotted line and is oriented 30 ˚ from the vertical axis. Principal stress orientations are shown as σ_1*'* and σ_3*'* resulting in effective normal and shear stress in the fault zone (σ_n*'* and τ respectively). Near fault full Wheatstone bridge strain gage is located 3 mm from the fault and records ε_1-ε_3 directly which allow direct estimation of σ_1- σ_3 through calibration during elastic loading phase of each experiment. **(b)** Cross-polarized optical microscope image of the intact granite (x40). WG is mostly composed of quartz, feldspars and in minor quantities of micas.

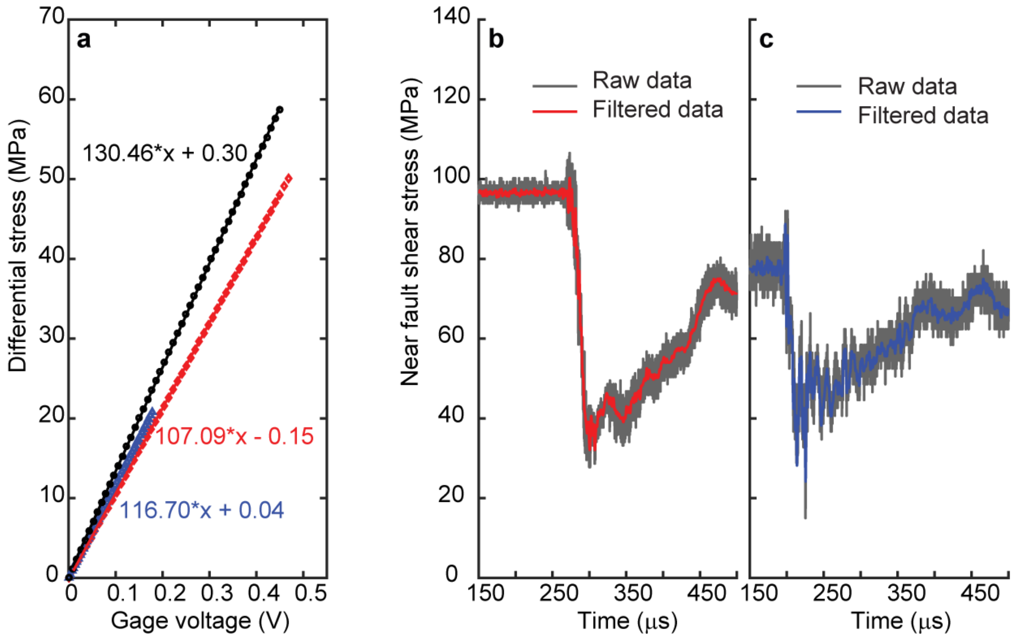

Supplementary Figure. 2. Strain gage calibration procedure and data filtering.

(a) Strain gage calibration. The strain gages were calibrated using low frequency stress measurements. Here, we assumed linear elasticity. Therefore, during the elastic loading phase of the experiments, since the young's modulus of the rock remained constant, we directly calibrated the differential strain recorded by the gages with the differential stress recorded by the far field sensors through a linear regression best fit. Since the linear elasticity assumption remained valid during the dynamic stick-slip events[9], at high sampling rate, the differential strain was also converted directly to differential stress. **(b)**, **(c).** Examples of data filtering procedure. Near fault shear stress evolution on time for one event in dry conditions **(b)** and in low fluid pressure conditions **(c)**. The grey trace accounts in both cases for raw data and the coloured lines account for the filtered data. The filter was set-up through identification of the noise frequencies present before the stress drops. Such frequencies were identified to be superior to 200 kHz so a low pass filter was applied to all the traces in order to enhance clarity in the presented figures without compromising the data.

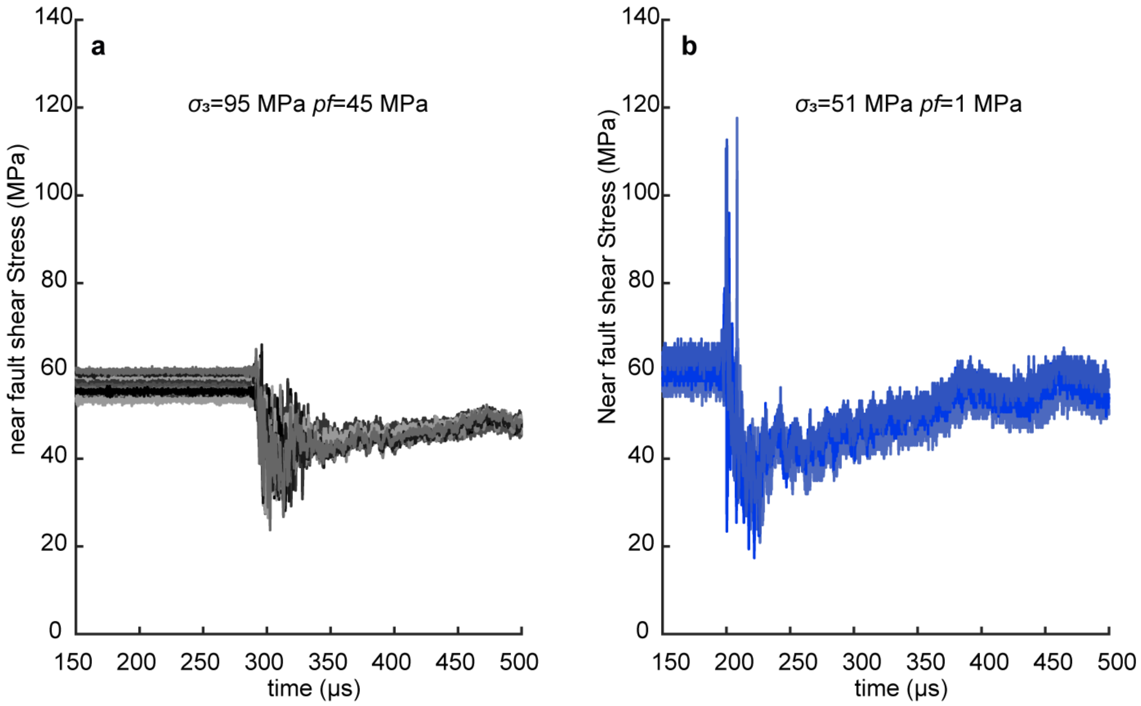

Supplementary Figure. 3. Dynamic shear stress evolution of additional experiments at 50 MPa effective confining pressure.

Dynamic shear stress evolution recorded at high sampling frequency (F_s =10.10^6 Hz). Each curve corresponds to one recorded laboratory earthquake. **(a)** Stick-slip events of high fluid pressure experiment at 95 MPa confining pressure and 45 MPa fluid pressure. In a similar way to the high fluid pressure experiment at *pf*=25 MPa experiment (Fig.1d), we observed low dynamic stress drops and no major increase in shear stress right before the event. **(b)** Stick-slip events of low fluid pressure experiment at 51 MPa confining pressure and 1 MPa fluid pressure, in a similar way to the low pore pressure experiment at *pf*=1 MPa experiment (Fig.1c), we observed extraordinarily high dynamic stress drops due to important increase in shear stress right before the dynamic stress drop. Such behaviour accounts for reproducibility of our experiments and was in perfect compliance with experiments performed at 70 MPa effective confining pressure, its microstructural observations, the suggested model and proposed interpretations.

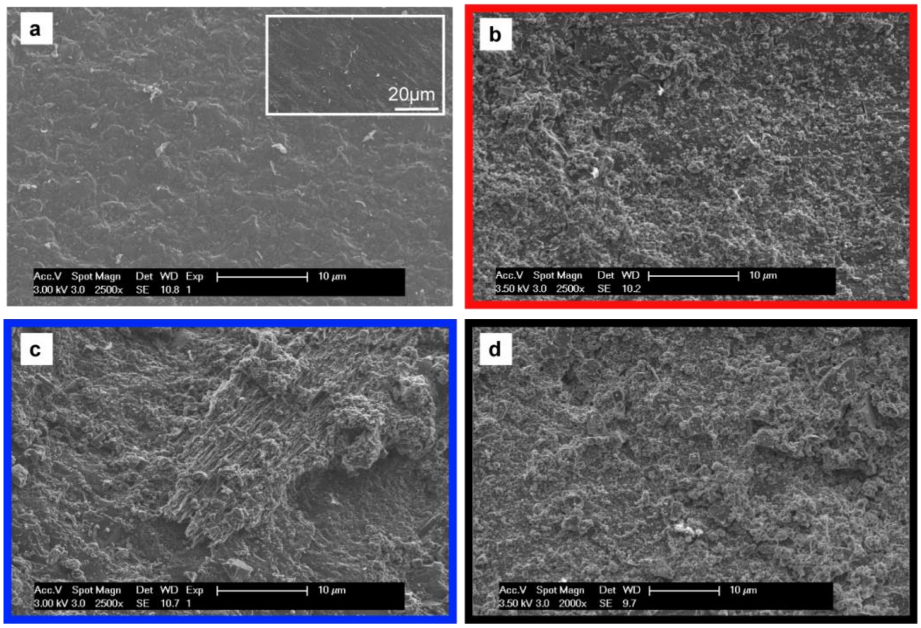

Supplementary Figure. 4. Low magnification micrographs of fault surfaces of experiments at σ_3'=70 MPa.

Scanning Electron Microscope images under secondary electron mode of the half-fault surfaces. Secondary electron mode allows observation of surface topography. **(a)** Undeformed sample surface. Initial surfaces were flat and homogeneous with asperity diameters ranging from 2 to 20 µm under no confinement. **(b)** Sheared surface of the room humidity experiment (dry conditions). The sheared surfaces were covered with clasts of sizes inferior to 5 µm. The clasts were found over and under ropy stretched material in the sense of shear. Glassy slabs resulted from cooling of the melted asperities. **(c)** Sheared surface of the low fluid pressure experiment (*pf* = 1 MPa). The sheared surfaces were again covered with clasts of sizes inferior to 5 µm. Patches covered with a layer of ropy like structures stretched in the shear direction were observed over and under the clasts. **(d)** Sheared surface of the high fluid pressure experiment (*pf*= 25 MPa). Again, clasts of sizes inferior to 5 µm were found all over the surface. This time no ropy-like structures were found, instead the surface presented mostly clasts of diameters ranging from .0.5 to 5 um evidence of brittle failure of asperities.

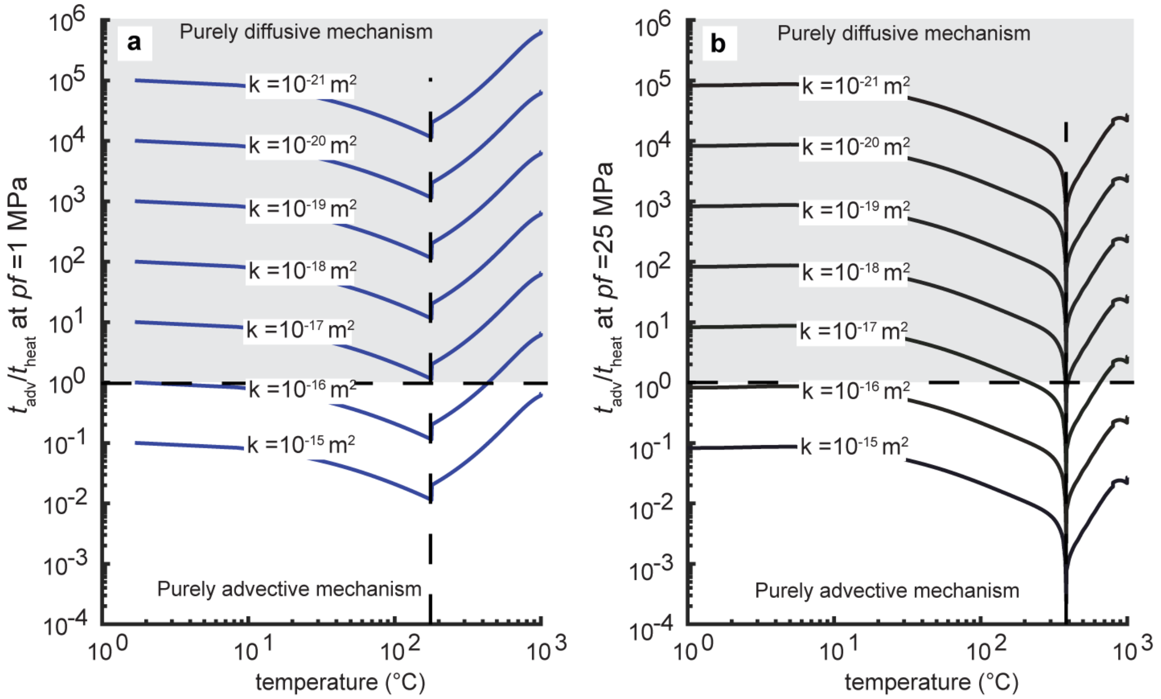

Supplementary Figure. 5. Heating regime of the interaction volume.

Calculation of the ratio between advection time (t_{adv}) and heat diffusion time (t_{heat}) in the interaction volume shown in Fig. 3b. **(a)** Calculations of the ratio t_{adv}/t_{heat} as a function of temperature for different fault permeabilities (k) considering the isobaric evolution with temperature of η, λ, ρ_w, and c_{pw} at 1 MPa fluid pressure[25]. **(b)** Similar calculations but this time considering isobaric evolution of the parameters at the experimental pressure of 25 MPa. The heating process is purely diffusive in the low pressure case (1 MPa pore fluid pressure) for fault permeabilities lower than 10^{-17} m^2 in the whole temperature range. At high fluid pressure (25 MPa); the process is purely diffusive for permeabilities lower than 10^{-18} m^2 but the cooling process should be enhanced by advection around the contacts at the temperature of the liquid/supercritical transition for fault permeabilities reaching 10^{-18} m^2.

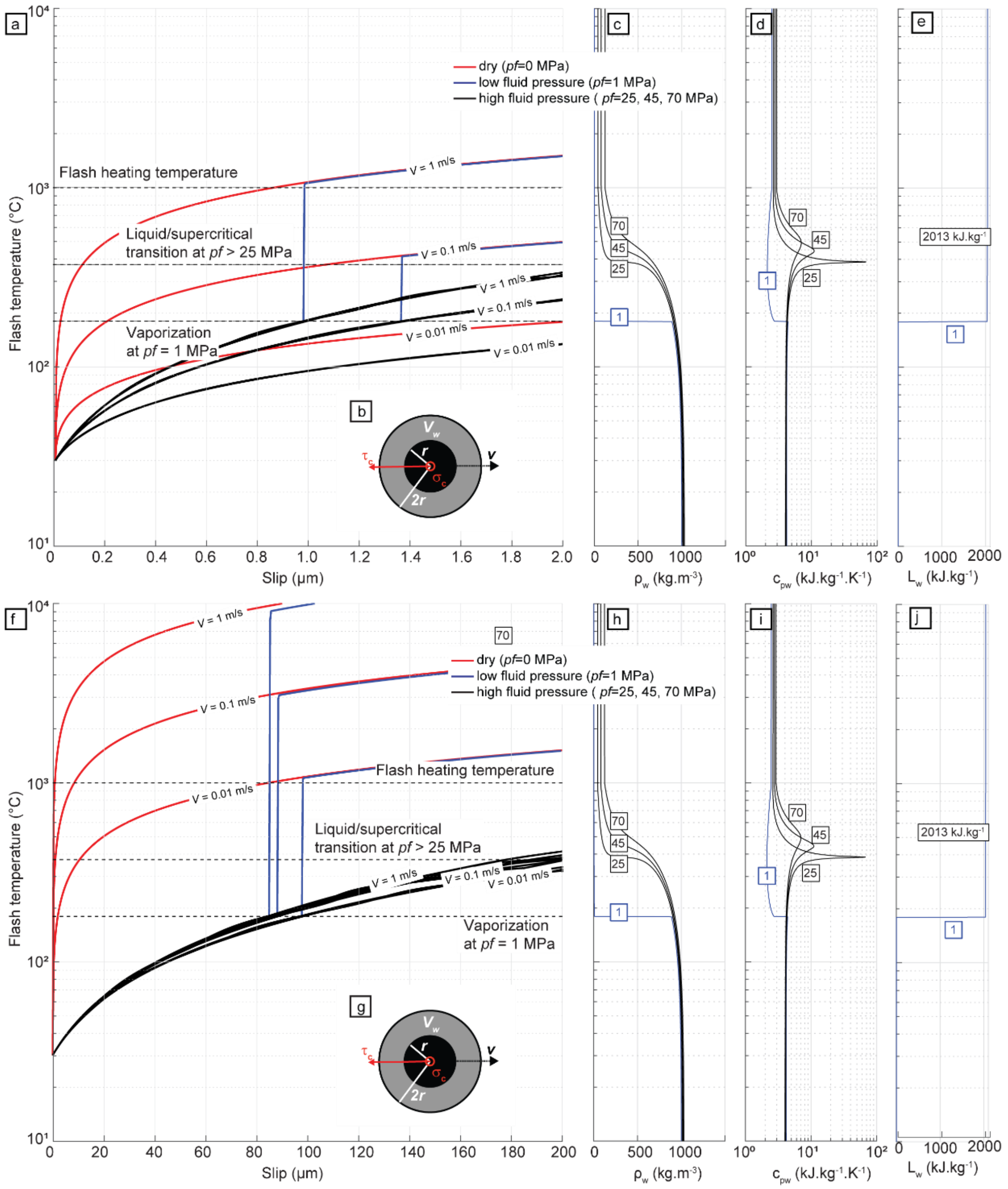

Supplementary Figure. 6. Flash temperature computation for other asperity sizes. **(a)**, **(b)**, **(c)**, **(d)**, **(e)** correspond to 2 μm asperities and **(f)**, **(g)**, **(h)**, **(i)** , **(j)** correspond to 200 μm asperities. The graphs for 20 μm asperities are shown in Figure 4 in the main text. Red curves correspond to dry conditions (*pf*=0 MPa), blue to low fluid pressure (*pf*=1 MPa) and black to high fluid pressure (full trace corresponds to *pf*=25 MPa and dotted traces to *pf*=45 and 70 MPa). **(a, f)** Flash temperature versus slip for different slip velocities. (v= 0.01, 0.1, 1 $m.s^{-1}$). **(b, g)** Schematic top view of the considered contact geometry **(c, h)** Temperature versus water density[25] each curve corresponds to the labelled pore pressure in MPa. **(d, i)** Temperature versus water's specific heat[25] each curve corresponds to the labelled pore pressure in MPa. **(e, j)** Temperature versus water's latent heat[25] each curve corresponds to the labelled pore pressure in MPa.

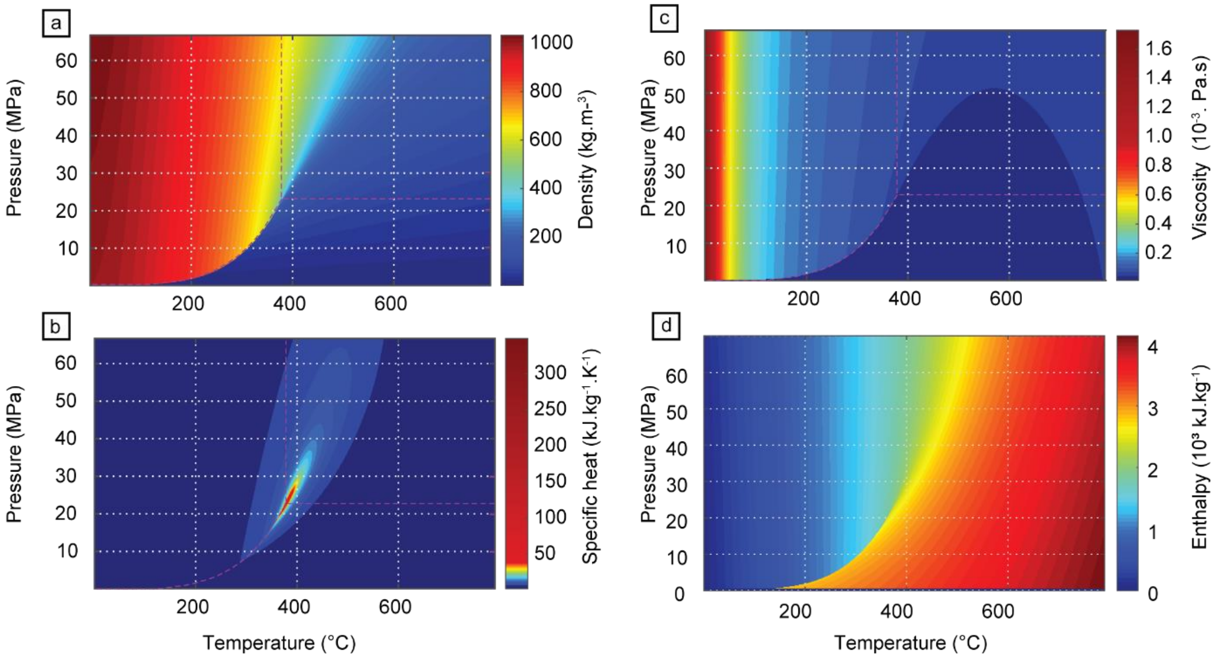

Supplementary Figure. 7. Thermophysical properties of water function of pressure and temperature.
(a) Water's density[25]. **(b)** Water's specific heat[25]. **(c)** Water's viscosity[25]. **(d)** Water's enthalpy[25].

Experiment	Event Number	Confining pressure	Fluid pressure	Effective confining pressure	τ_0	τ_p	τ_d	τ_f	σ_{n0}'	σ_{nf}'	Slip
name	#	MPa	MPa	MPa	MPa	MPa	MPa	MPa	MPa	MPa	µm
WG0_0	1	70	0	70	87,60	89,07	30,68	59,14	120,68	104,93	176,88
WG0_0	2	70	0	70	100,31	101,75	25,65	64,25	127,91	108,14	231,12
WG0_0	3	70	0	70	107,13	109,22	26,96	66,47	131,83	109,25	260,00
WG0_0	4	70	0	70	113,68	116,23	25,08	68,21	135,63	110,61	285,87
WG0_0	5	70	0	70	113,20	114,73	31,32	67,79	135,36	110,22	279,98
WG0_0	6	70	0	70	111,10	115,13	24,07	65,72	134,16	109,17	258,81
WG0_0	7	70	0	70	106,05	109,16	24,41	64,97	131,25	108,78	265,41
WG0_0	8	70	0	70	102,37	104,60	28,32	64,47	129,13	108,56	237,97
WG0_0	9	70	0	70	99,17	105,98	26,81	63,65	127,28	107,94	237,73
WG0_0	10	70	0	70	97,01	104,18	30,24	63,03	126,05	107,49	225,08
WG1_1	1	71	1	70	65,04	71,94	12,33	54,44	106,92	101,10	164,33
WG1_1	2	71	1	70	71,21	120,55	16,25	58,16	110,48	103,33	181,82
WG1_1	3	71	1	70	77,72	92,68	14,00	62,13	114,23	105,65	201,14
WG1_1	4	71	1	70	83,99	112,99	19,26	66,25	117,83	108,03	210,55
WG1_1	5	71	1	70	91,27	118,38	1,53	59,88	122,04	104,53	254,38
WG25_1	1	95	25	70	52,14	54,40	40,01	48,61	100,19	98,23	29,48
WG25_1	2	95	25	70	53,36	59,41	42,62	49,13	100,91	98,55	31,84
WG25_1	3	95	25	70	54,41	58,18	43,47	50,64	101,53	99,45	31,35
WG25_1	4	95	25	70	57,94	61,66	44,62	52,51	103,54	100,51	40,30
WG25_1	5	95	25	70	58,68	61,01	42,50	51,97	103,98	100,20	39,41
WG25_1	6	95	25	70	59,94	62,31	42,73	55,26	104,70	102,13	46,14
WG25_1	7	95	25	70	62,66	67,20	46,68	55,95	106,29	102,54	45,07
WG25_1	8	95	25	70	62,17	63,85	45,84	56,38	106,03	102,77	33,16
WG25_1	9	95	25	70	61,73	65,30	44,68	58,07	105,77	103,75	33,32
WG25_1	10	95	25	70	64,96	70,11	45,44	57,42	107,60	103,41	49,25
WG25_1	11	95	25	70	66,70	72,00	49,08	58,03	108,60	103,77	64,09
WG25_1	12	95	25	70	67,35	71,64	44,23	61,90	109,00	105,96	56,90
WG25_1	13	95	25	70	70,88	76,12	42,08	57,35	111,01	103,45	77,86
WG25_1	14	95	25	70	71,74	81,85	43,58	61,87	111,50	106,10	92,78
WG25_1	15	95	25	70	80,00	86,95	45,45	64,86	116,27	107,88	99,07
WG25_1	16	95	25	70	82,77	84,95	46,14	64,17	117,86	107,50	104,04

Supplementary Table 1. Experimental data presented in the main text.

Experimental data from stick-slip experiments presented in the main text of the manuscript. A total of 52 stick-slip events are presented under different confining and fluid pressures.

Parameter name	Symbol	Unit	Value	Remarks	Reference
Rock thermal diffusivity	κ	$m^2.s^{-1}$	1,00E-06	WG thermal diffusivity	(7)
Rock density	ρ	$kg.m^{-1}$	2650	WG density	(7).
Rock specific heat	c_p	$J.kg^{-1}.K^{-1}$	830	WG specific heat	(7).
Asperity radius	r	µm	20	Determined from microstructural observations, other asperity sizes in Extended Data	
Asperity height	h	µm	20		
Volume of water involved in cooling	Vw	µl		Vw=h*π*((2r)^2-r^2)	(7).
Indentation hardness	Pm	MPa	6000	Estimated WG indentation hardness	(32).
Peak static shear stress	$\tau 0$	MPa	70	value taken from the low pore pressure experiment at 70 MPa effective confining pressure	-
Real contact area/nominal contact area	α	-	60	α=Ar/A=Pm/σn0	(30).

Supplementary Table 2. Flash temperature computation parameters

Parameters used in the flash temperature model modified for fluid pressures presented in the main text.

Parameter name	Symbol	Unit	Value	Remarks	Reference
Rock thermal diffusivity	κ	$m^2.s^{-1}$	1,00E-06	WG thermal diffusivity	(7).
Rock density	ρ	$kg.m^{-1}$	2650	WG density	(7).
Rock specific heat	c_p	$J.kg^{-1}.K^{-1}$	830	WG specific heat	(7).
Fault permeability	k	m^2	1E-17	Fault permeability	
Fault Porosity	φ	-	0,3	Fault's porosity	(15).
Slip Velocity	V	$m.s^{-1}$	1	Seismic slip velocity	(1, 2).
Initial friction	f	-	0,7	Static friction coefficient	(1).
Experimental Shear stress	τ	MPa	70	Value representative of our pore fluid experiments	
Solid expansivity	λ_n	$^\circ C^{-1}$	-0.19e-3	Solid Thermal expansivity from Rice, 2006	(2).
Solid compressibility	β_n	Pa^{-1}	6,50E-10	Solid Thermal compressibility from Rice, 2006	(2.)
Fluid expansivity	λ_f	$^\circ C^{-1}$		Interpolated from NIST	(25).
Fluid compressibility	β_f	Pa^{-1}		Interpolated from NIST	(25).
Fluid dynamic viscosity	η	$Pa.s^{-1}$		Interpolated from NIST	(25).
Fluid density	ρ_w	$kg.m^{-3}$		Interpolated from NIST	(25).
Fluid specific heat	c_{pw}	$J.kg^{-1}.K^{-1}$		Interpolated from NIST	(25).

Supplementary Table 3. Bulk fault temperature computation parameters

Parameters used in the finite difference temperature model modified for fluid pressures presented in the main text.
